# Nanovaccines against Cervical Cancer: Reliable Strategies to Circumvent Limitations of Traditional Therapeutic Vaccines

**DOI:** 10.34172/apb.43712

**Published:** 2025-03-08

**Authors:** Enwa Felix Oghenemaro, Safia Obaidur Rab, Ebraheem Abdu Musad Saleh, Asmaa F. Kassem, Jasur Rizaev, Deepak Nathiya, Parjinder Kaur, M. Ravi Kumar, Karam Kadhim, Ahmed M. Hashim

**Affiliations:** ^1^Department of Pharmaceutical Microbiology, Faculty of Pharmacy, Delta State University, Abraka, Delta State, Nigeria.; ^2^Department of Clinical Laboratory Sciences, College of Applied Medical Science, King Khalid and University, Abha, Saudi Arabia.; ^3^Department of Chemistry, College of Science and Humanities in Al-Kharj, Prince Sattam Bin Abdulaziz University, Al-Kharj, 11942, Saudi Arabia.; ^4^Chemistry of Natural and Microbial Products Department, Pharmaceutical and Drug Industries Research Institute, National Research Centre, Dokki, Cairo 12622, Egypt.; ^5^Department of Public Health and Healthcare Management, Rector, Samarkand State Medical University, 18, Amir Temur Street, Samarkand, Uzbekistan.; ^6^Department of Pharmacy Practice, NIMS Institute of Pharmacy, NIMS University Rajasthan, Jaipur, India.; ^7^Chandigarh Pharmacy College, Chandigarh Group of Colleges-Jhanjeri, Mohali 140307, Punjab, India.; ^8^Department of Basic Science & Humanities, Raghu Engineering College, Visakhapatnam, India.; ^9^Medical Laboratory Techniques Department, College of Health and Medical Techniques, Al-Mustaqbal University, Babylon, Iraq.; ^10^Mazaya University College, Dhiqar, Iraq.

**Keywords:** Cervical cancer, Therapeutic vaccine, Nanotechnology, Immune response, Nanovaccine

## Abstract

Cervical cancer ranks fourth in terms of diagnosis and cancer-related deaths in women worldwide. Despite the approval of prophylactic vaccines against cervical cancers, these vaccines are not able to eradicate the existing ones. Therefore, various platforms have been developed to design therapeutic vaccines against cervical cancers, including DNA/RNA-based, protein/peptide-based, vector-based, and cell-based platforms. Despite the advantages of each platform, therapeutic vaccines have displayed limited clinical benefit in patients with cervical cancer, which is partially associated with inefficient delivery of vaccine components. To address these issues, different nanoplatforms have been developed to carry cellular or molecular components of vaccines to target cells and lymphoid tissues, thus promoting the durability and potency of immune responses against tumor cells and antigens besides decreasing side effects. Moreover, nanoparticles (NPs), as adjuvants and/or carriers, provide other advantages, including sufficient antigen loading and uptake by antigen-presenting cells (APCs), adaptable antigen presentation, high immunogenicity, high stability, increased lymph node retention, and precise targeting. Thus, nanovaccines also lead us to design and develop personalized vaccines against cervical cancer. Here, we discuss platforms that have been used in clinical trials for the treatment of cervical cancer, their advantages and disadvantages, platforms for developing nanovaccines, and how they improve the therapeutic efficacy of vaccines.

## Introduction

 Cervical cancer is ranked fourth after breast, colorectal, and lung cancers in occurrence (604 000 new cases, 6.5%) and fourth in regards to cancer-related deaths (342 000 deaths, 7.7%) in women.^1^ Although infection with human papillomavirus (HPV) is the main risk factor,^2^ other cofactors, including long-term use of oral contraceptives, increased births, smoking, and several sexually transmitted diseases (*Chlamydia trachomatis* and HIV), also are involved in the development of cervical cancer.^3^ Genital HPV types are divided into low-risk (non-carcinogenic) and high-risk (carcinogenic) according to their association with precursor lesions and malignant forms of cervical cancer. There is evidence that persistent infection with high-risk HPVs is responsible for approximately 99.7% of cervical malignancies.^4^ HPV contains about 8 kb of double-stranded and circular form DNA which has eight open reading frames, including early genes (E1, E2, E4, E5, E6, and E7) which are involved in the replication of the viral DNA and late genes (L1 and L2) which encode structural proteins, such as capsid.^5,6^ Two early genes, E6 and E7, act as oncogenes and produce their oncoprotein forms which promote the degradation of tumor suppressor genes p53 and retinoblastoma protein (pRb), respectively, leading to dysregulation in signaling pathways and cellular proliferation.^6^

 In addition to disappointing outcomes, a narrow therapeutic window and serious side effects are other challenges of systemic chemotherapeutic agents.^7^ Despite the approval of three prophylactic vaccines against L1/L2 capsid antigens, these vaccines are not effective against established tumors because L1/L2 capsid antigens are not expressed at appreciable levels in cervical cancer.^8^ Therefore, therapeutic strategies, such as various new immunotherapeutic approaches, are currently under investigation to enhance treatment outcomes in cervical cancer patients. One of the immunotherapy approaches in the treatment of cervical cancer is the development of therapeutic vaccines based on HPV oncogenes, E6 and E7.^9^ The presence of immune escape mechanisms as well as low immunogenicity of tumors and heterogeneity decline the therapeutic efficacy of cancer vaccines.^10^ Furthermore, each therapeutic vaccine platform has specific limitations: lower delivery efficiency and mutagenesis risk for DNA-based vaccines, lower immunogenicity and requiring adjuvants for peptide-based vaccines, higher cost and complex preparation process for dendritic cell (DC)-based vaccines, and potential pre-existing immunity and safety concerns for viral/bacterial-based vaccines.^11^ Given the limitations of traditional therapeutic vaccines, the field of nanotechnology has emerged as a promising solution, providing innovative platforms for vaccine delivery and efficacy enhancement.^12,13^ In these systems, nanoparticles (NPs) activate immune responses against the specific antigen by carrying and delivering antigens and immunomodulators to the target cells. Regarding vaccine development, nanostructures can protect adjuvants and antigens from degradation, co-deliver adjuvants and antigens to antigen-presenting cells (APCs) and tumor cells to enhance their immunogenicity, and prolonged antigen release and their exposure to the immune system to elicit strong responses.^14^ These formulations are called nanovaccines. The modification of NPs’ surface or changing their surface properties facilitates targeting and delivering nanovaccine cargoes to APCs and lymphoid tissues.^15,16^ Nanovaccines also offer size and shape advantages for precise controlling and adapting to various cargos.^17^ It is worth noting that some NPs have the characteristics of immune adjuvant, providing not only a carrier system for antigens, but also augments immune responses.^18^ Therefore, due to the disability of the approved prophylactic vaccines against established HPV infections and challenges of conventional therapeutic vaccines against HPV-induced cervical cancer, nanovaccines have been developed to mitigate these drawbacks. In this paper, we will discuss various platforms of therapeutic vaccines in the treatment of cervical cancer, their advantages and disadvantages, and their application in clinical trials. Finally, the main focus of our paper will be on several forms of nanovaccines against cervical cancer.

## Cancer vaccines

 Since the discovery of the first vaccine by Edward Jenner in 1796, the development of vaccines has reached great achievements in preventing many infectious diseases through stimulating immune cells and immune responses to specifically and rapidly clear or control invading pathogens and thereby prevent disease. Regarding cancer, the use of vaccines is noticeably an extension of their preventive application, but challenges to achieving the therapeutic ones is a frustrating journey.^19^ In addition to Cervarix, Gardasil, and Gardasil 9 have been approved as prophylactic vaccines against HPV and HPV-induced cervical cancers, Heplisav-B is also approved to prevent hepatitis B virus (HBV)-induced hepatocellular carcinoma and liver cancer.^20^ Regarding prophylactic vaccines against HPV infection, they have limited impact on established infections and fail to elicit robust CD4^+^ and CD8^+^ T-cell responses critical for controlling precancerous and cancerous lesions.^21^ Moreover, limiting vaccination owing to the high prices of vaccines and inadequate population coverage results in a substantial population remaining afflicted with high-risk HPV infections and their associated pathologies.^22^ Therefore, needing for vaccines, called therapeutic vaccines, to elicit immune responses against existing HPV infections and HPV-induced cervical cancers is crucial. Therapeutic cancer vaccines usually involve the administration of selected tumor-associated antigens (TAAs) or tumor-specific antigens (TSA) in combination with adjuvants that activate APCs, leading to the stimulation of the adaptive immune system of patients to control the growth of tumors. TAAs are antigens that are overexpressed or abnormally expressed in tumor cells, but may also be present in normal cells. In contrast, TSAs are strictly expressed by tumor cells and are not found in normal tissues.^23^ The primary fundamentals required for reliable outcomes of therapeutic vaccination against tumors include delivery of high-quality and large amounts of TAAs or TSAs to APCs, optimum activation of APCs, and robust and persistent activation of CD4^+^ T helper cells and cytotoxic T lymphocytes (CTLs).^24^ TAAs refer to antigens either abnormally or preferentially expressed on tumor cells, while normal cells also express them at some level.TSAs are not expressed on normal cells and are strictly tumor-specific, which include antigens from oncoviruses (such as HPV E6 and E7 antigens) and mutated neoantigens.^25,26^ Development of therapeutic cancer vaccines faces three main challenges compared with prophylactic vaccines: (1) established malignancy, (2) an immunosuppressive tumor microenvironment (TME), and (3) low immunogenicity.^25^ Despite these limitations, two therapeutic vaccines have been licensed for the treatment of cancers: Bacillus Calmette-Guérin (BCG) vaccine, a live attenuated strain of *Mycobacterium bovis*, for patients with early-stage bladder cancer and Sipuleucel-T (Provenge®) vaccine, a DC-based vaccine, for prostate cancer.^27^ Therefore, attempts to develop therapeutic vaccines are hopeful and scientists investigate various strategies and platforms to introduce reliable and effective cancer vaccines. However, the development of therapeutic cancer vaccines faces challenges, including ensuring the delivery of vaccine components to the TME or secondary lymph organs, storage and stability of vaccines, co-delivery of adjuvant and antigen to the same APC, using appropriate adjuvants, and lower efficiency as a monotherapy.^28,29^ Nanotechnology and nano-sized structures address these challenges by targeting the TME and APCs, protecting vaccine components from degradation, controlling antigen and adjuvant release and their distribution, co-delivery of antigen and adjuvant to the same APC, and providing combination ability for a vaccine with other therapeutic agents.^30^ DCs are crucial players in stimulation of anti-tumor immune responses owing to their important role in the presentation of antigens and priming CD8^+^ T-cells against antigens, thus, targeting DCs using nanovaccines augments antigen-specific immune responses. Incorporation of antibodies and ligands on NPs to target molecules on the DCs, such as c-type lectin receptors (CLRs), Clec9a or DNGR-1, mannose receptor (MR) or CD206, and DEC-205 (CD205), is a reliable strategy in nanovaccine development.^14^ For example, Saluja et al coated the surface of an antigen-encapsulated poly lactic-co-glycolic acid (PLGA) with anti-DEC-205 to target DEC-205-expressing DCs and increase antigen delivery efficiency to them. This targeting system bypassed the need for the classical presentation and directly accessed the class I cytoplasmic MHC loading machinery, leading to a remarkable increase in DC stimulation of anti-tumor CD8^+^ T-cells.^31^ In another study, Meng et al designed a lipid-coated iron oxide NP to encapsulate peptide antigen and CpG DNA, as an adjuvant, and deliver them into cytosol and lysosomes of DCs. The developed nanovaccine not only accumulated in the DCs of draining lymph nodes and promoted DC maturation, but also increased the population of the antigen-specific T-cells in the spleen and tumor, leading to improved animal survival and inhibited tumor growth.^32^ Nanocarriers in nanovaccine constructs also can stimulate immune responses. For instance, bacterial outer membrane vesicles (OMVs) could stimulate toll-like receptor 4 (TLR4) and TLR5 owing to containing immunostimulating signals lipopolysaccharide (LPS) and flagellin, respectively, suggesting them as carriers and adjuvants.^33^ Therefore, nanovaccines have been developed to overcome the limitations of conventional therapeutic vaccines.

## Therapeutic vaccines against cervical cancer in clinical trials

 According to the platforms, therapeutic vaccines against cervical cancers can be divided into four classes: DNA/RNA-based vaccines, protein/peptide-based vaccines, vector-based vaccines, and cell-based vaccines (Table 1). Regarding nucleic acid-based vaccines, a direct translation of mRNA molecules occurs in the cytoplasm, whereas DNA passes an additional step to enter the nucleus. This may increase the potential risk of mutagenesis because of the integration of DNA into the host chromosome.^27^ Despite the relatively easy manufacture of nucleic acid-based vaccines, their development and application face some challenges. For instance, strong negative charge and high molecular weight prevent the passive diffusion of these biomolecules across the cellular membranes.^34^ Despite the facile production and storage, safety, and stability, protein/peptide-based vaccines require adjuvants to elicit potent immune responses. The limitation of vector-based vaccines is related to their safety and effectiveness problems due to pathogenicity and a stronger immune response to vectors than their corresponding antigens, respectively.^22^ Moreover, the application of cell-based vaccines is limited because of the high cost of manufacturing and high mortality before arriving at the lymph nodes.^35^ Despite these limitations, several clinical trials using the platforms have been conducted against HPV-induced cervical cancer.

**Table 1:** Clinical trials with different vaccine platforms for cervical cancerous and precancerous diseases

**Platform**	**Vaccine name**	**Phase**	**Status**	**NCT number**
DNA-based	VB10.16	I/II	Completed	NCT02529930
	GX-188E	II	Unknown	NCT02596243
	VGX 3100	II	Completed	NCT01304524
	pNGVL4a-Sig/E7(detox)/HSP70	I/II	Completed	NCT00121173
RNA-based	BNT113	I/II	Recruiting	NCT03418480
Protein-based	HspE7	II	Completed	NCT00054041
	TA-CIN	I	Active, not recruiting	NCT02405221
	SGN-00101	II	Completed	NCT00091130
	ProCervix	II	Completed	NCT01957878
Peptide-based	DPX-E7	I/II	Active, not recruiting	NCT02865135
	PepCan	II	Completed	NCT02481414
	ISA101/ISA101b	I/II	Completed	NCT02128126
	ISA101	II	Completed	NCT02426892
Viral vector-based	RO5217790	II	Completed	NCT01022346
	TA-HPV	II	Completed	NCT00002916
	HB-201	I/II	Recruiting	NCT04180215
Bacterial vector-based	ADXS11-001	III	Terminated	NCT02853604
	ADXS11-001	II	Completed	NCT01266460
Dendritic cell-based	DC vaccine	I	Unknown	NCT03870113
	DC vaccine	I	Unknown	NCT00155766
Other cell-based	E7 TCR	I/II	Recruiting	NCT02858310
	BVAC-C	I/II	Completed	NCT02866006

**Figure 1:**
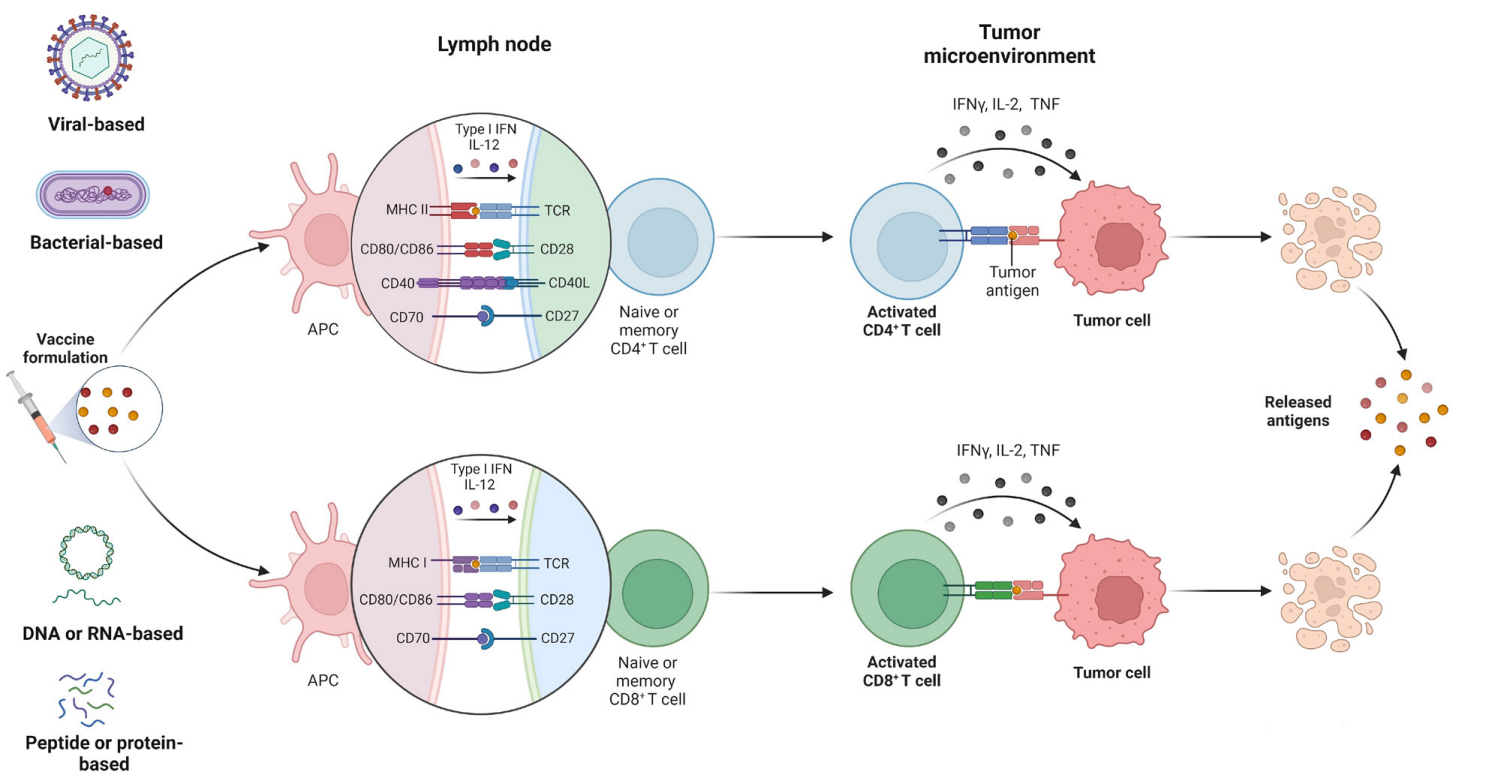
Therapeutic vaccines in various formulations elicit both CD4+ and CD8+ T-cell responses against tumor cells.

 A Phase I study using a DNA vaccine targeting E6 and E7 of HPV-16/18 with IL-12 encoding plasmid, called MEDI0457 (INO-3112), was conducted on 10 patients with HPV16- and HPV18-induced cervical cancers in which patients received MEDI0457 (1 mg of INO-9012 and 6 mg of VGX-3100) by electroporation (EP) every 4 weeks for a total of 4 doses following chemoradiation or radiation alone and followed up for at least 6 months after last vaccination. The study revealed that intramuscularly administration of MEDI0457 following chemoradiation generates IFNγ-producing T-cell and anti-HPV antibody responses and decreases PD-1^+^CD8^+^, PD-L1^+^CD8^+^, and PD-L1^+^CD68^+^ subpopulations with cleared detectable HPV DNA in cervical biopsy specimens. Also, they reported that 8 of 10 patients exhibited treatment-related adverse effects in which only injection site pain and injection site bruising were reported in more than 1 patient, suggesting that the vaccination strategy was well-tolerated.^36^ Regarding vector-based vaccines, a study showed that oral vaccination with NZ8123-HPV16-optiE6 (n = 32), a vaccine based on *L. lactis*, induced long-term E6-specific IFN-γ-secreting CD8^+^ CTL responses without serious adverse effects, whereas humoral responses decreased after 6 months of the last vaccination. The most common vaccine-related adverse effects were nausea and vomiting at mild to moderate intensity.^37^ Furthermore, the safety and efficacy of a live-attenuated Listeria monocytogenes vaccine, called ADXs11-001 or Lm-LLO-E7, were assessed in phase I (n = 15) and phase II (n = 109) studies at doses of 1 × 10^9^, 3.3 × 10^9^, and 1 × 10^10^ colony-forming units (CFUs).^38,39^ In phase I, a flu-like syndrome was reported in all patients and 40% of patients experienced grade 3 adverse effects, including pyrexia, fatigue, and increased gamma-glutamyl transferase (GGT), while no grade 4 adverse effects were reported.^38^ Intravenous administration of the ADXs11-001 vaccine + cisplatin promoted HPV16 E7-specific T-cell responses and improved survival rates in patients with cervical cancer with acceptable safety.^39^ In a phase II study, Rebucci-Peixoto et al found that the combination of UCPVax (1 mg, subcutaneously), composed of two separate peptides derived from human telomerase reverse transcriptase (hTERT), with atezolizumab (1,200 mg, intravenously) could activate and promote antitumor T-cell immunity in patients with HPV^+^ cancer.^40^ Figure 1 summarizes how therapeutic vaccines induce immune responses against tumor cells.

## Nanovaccines against cervical cancer

**Figure 2:**
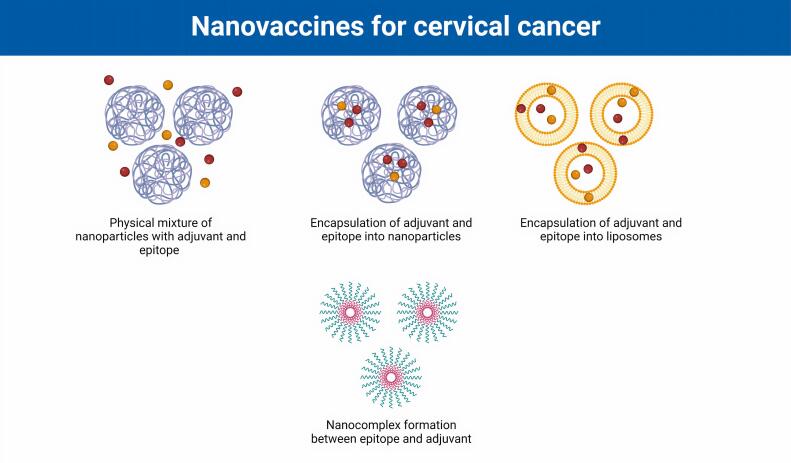
Various formulations of nanovaccines against cervical cancer.

 Despite great efforts in the design and development of therapeutic vaccines against cervical cancer, there are not yet any FDA-approved vaccines on the market. Some challenges limit the application of conventional platforms for cancers, including the induction of short-term immune responses, weak immunogenicity, poor biocompatibility and stability, and inefficient vaccine delivery.^41^ Indeed, protein antigens are moderately fragile and readily degraded in the blood microenvironment, leading to reduced delivery to cells and unsuccessful immunity.^42^ Therefore, designing and constructing therapeutic vaccines using novel technologies, such as nanotechnology, could be effective. In general, various nanoformulation strategies have been used for the development of nanovaccines for the treatment of cervical cancer, such as multiple forms of NPs and liposomes for the encapsulation of antigens and adjuvants as well as the formation of nanocomplexes between vaccine components (Figure 2).

 An ideal vaccine candidate should be able to promote immunological memory and address immune tolerance. Since T-cell activation and memory cell differentiation occur following antigen capture by APCs, such as DCs and macrophages, and their migration into the secondary lymphoid organs, such as lymph nodes and spleen, targeting lymphatic organs with nanovaccines improves vaccine efficacy via generating long-term immunological memory.^43^ For instance, Xiao et al designed a nanovaccine in which imiquimod (R@837)-loaded PLGA NP were capped with the antigenic cancer cell membrane (CCM) (called CCMP@R837). The CCMP@R837 nanovaccine stimulated tissue-resident memory T-cells (T_RM_) following enhanced DC uptake to secrete cytokines, such as IL-12. The matured DCs activate the naïve CD8^+^ T-cells and suppress the differentiation of naïve CD4^+^ T-cells to Treg, leading to their proliferation and differentiation into central memory T-cells (T_CM_) and effector memory T-cells (T_EM_). The generated long-term immunity could recognize and destroy cancer cells.^44^ Similarly, Luo et al indicated that decorating the surface of NPs using cancer cell membrane proteins and mannose to specifically target lymph nodes and DCs not only promotes robust antigen-specific CD8^+^ T-cell responses, but also elicits memory T-cells.^45^ It is worth noting that memory CD8^+^ T-cells are classified into two classes: (1) T_CM_ which are localized in the lymph nodes and are CD3^+^ CD8^+^ CD62L^+^ CD44^+^ and (2) T_EM_ which are found in non-lymphoid organs and are CD3^+^ CD8^+^ CD62L^-^ CD44^+^. ^46^ Therefore, designing nanovaccines for targeting and priming lymph nodes as well as enhancing DC uptake to elicit memory T-cells will be hopeful in cancer therapy. In addition to their critical role in immunity, DC targeting in the development of nanovaccines is pivotal owing to their ability to control immune tolerance.^47^

###  Nanoparticles

 Nanomaterials and NPs are hopeful delivery systems for cancer vaccines. Several nanocarriers have been used to target and deliver antigens or adjuvants to specific cells by modifying their surface composition and/or properties and to enhance both innate and adaptive immune responses, such as adjuvants.^48,49^ Besides their delivery application, NPs are already used for cancer prevention and treatment by efficiently inducing sustain immune responses. Some parameters determine the effectiveness of NPs in promoting immune responses, including shape, surface characteristics (hydrophilicity, charge, etc.), particle size, kinetics, etc. Various types of NPs have been used as delivery vehicles for antigens and adjuvants or acted as adjuvants, including polymeric NPs (synthetic and natural), metal and metal-oxide NPs, dendrimer NPs, solid lipid NPs, carbon nanotubes, so on.

 Polymeric materials are the most common substances for fabricating NP-based drugs, consequently, vaccine, carriers, which are classified as natural and synthetic polymers. Natural polymers, such as albumin, chitosan, hyaluronic acid, dextran, inulin, and alginate, obtained from natural, sustainable, and renewable resources with biodegradability, biocompatibility, and non-toxic properties, proposing them as promising candidates in vaccine formulation. PLGA, poly(lactic acid) (PLA), poly(ethylenimine) (PEI), and poly(ε-caprolactone) (PCL) are the main synthetic polymers that have been used in vaccine development. Due to high safety profiles and excellent controllable biodegradability, biocompatibility, and erosion, two synthetic polymers, PLGA and PLA, are approved by FDA for biomedical applications. In addition to polymeric NPs, metal and metal-oxide NPs are another class of promising NPs used for the development of therapeutic vaccines owing to precise control and modification of their charge, shape, size, and surface, as well as optical properties and higher density. A variety of metallic NPs and their oxide forms have been used as delivery systems in cancer vaccines, such as aluminum, gold, silver, zinc, titanium, cobalt, cuprous, and iron.^50^ It is worth noting that the transport and uptake of NPs and subsequently the release of antigen and/or adjuvant can be affected by pH, temperature, and metabolites of APCs. Therefore, constructing smart NPs sensitive to cellular conditions could be considered in their designing. Despite great achievements, NPs and NP-based nanovaccines have some challenges. Firstly, the mononuclear phagocyte system and reticuloendothelial system could easily recognize and eliminate NPs before they can reach the target tissue and exert their therapeutic effects, therefore, using coating strategies and biomimetic materials could tackle this problem.^51^ Secondly, the off-target accumulation of NPs in other organs as well as the non-degradable natures of some NPs, such as metal-based NPs, raise safety concerns, called nanotoxicology, leading to oxidative stress and mitochondrial dysfunction, lysosomal damage, DNA damage, and cell death.^52^ The large-scale production with a high level of consistency and in a sterile condition for application in clinical trials is another challenge in using NPs.^53^

 Regarding cervical cancer, several studies investigated the application of various NPs as carriers of antigens and adjuvants. For instance, Rahimian et al assessed the efficacy of hydrophilic polyester (poly(d,l lactic-co-hydroxymethyl glycolic acid) (pLHMGA)) NPs-loaded with HPV16 E7 synthetic long peptide (SLP) and poly IC, a TLR3 ligand. At first, they characterized NPs according to their morphology, size, and zeta-potential using transmission electron microscopy (TEM), dynamic light scattering (DLS), and Zetasizer. To develop a mouse model of HPV-induced cervical cancer, they subcutaneously inoculated TC-1 cell lines and subsequently vaccinated mice when the tumors were palpable and at day 21 as a boost. The administration of encapsulated E7 SLP and poly IC NPs, with 491 nm particle size and -25 mV surface charge, expanded HPV-specific CD8^+^ T-cells and reduced tumor growth in tumor-bearing mice. Also, the administration of pLHMGA exhibited a safe profile without adverse effects.^54^ In another study, Zhang et al used PLGA NPs for the encapsulation of the antigenic peptide HPV16 E7 and an adjuvant, adenosine triphosphate (ATP). ATP performs as an adjuvant owing to its ability to induce immune responses by acting as an endogenous extracellular danger signal. They reported that PLGA NPs enhanced the stability of E7 peptide and increased its accumulation in lymph nodes and DCs uptake, in which ATP promoted DCs migration and maturation. The nanovaccine not only displayed therapeutic efficacy by inhibiting tumor growth, but also exhibited prophylactic activity through the production of long-lasting immunity against tumor rechallenge. In a mechanistic manner, the constructed nanovaccine diminished the generation and infiltration of immunosuppressive cells, including myeloid-derived suppressor cells (MDSCs) and Treg cells, and improved the generation of CD8^+^ IFN-γ^+^ T-cells.^55^ Mardani et al used Pep-1, a short amphipathic peptide carrier, for delivery of the full-length HPV16 E7 protein into mammalian cells. The noncovalent binding between Pep-1 and E7 formed stable NPs induced higher levels of Th1 cellular immune response with the chief IFN-γ and IgG2a levels compared with E7 protein in a murine tumor model.^56^ To use the ability of peptides for constructing NPs, Zhao et aldesigned a therapeutic and prophylactic vaccine on E7- and L2-specific epitopes inserted on the surface of hyper-stable thioredoxin (Trx) scaffold and then converted into an NP using a heptamerization-promoting module (OVX313). The formulated nanovaccine promoted both B-cell immunity and CTL responses and regressed tumor growth in grafted TC-1 tumors in mice.^57^ It has been shown that targeting NPs toward APCs could enhance their therapeutic activities. For example, Rosalia et al directed PLGA-based nanovaccines toward DCs by targeting CD40. Firstly, they characterized the prepared various formulated NPs with zeta-potential and dynamic light scattering measurements and quantified encapsulated antigen and adjuvant. To analysis of the PLGA NP-uptake by immune cells *in vitro* and *in vivo*, cultured DCs were incubated with CD40-targeted or non-targeted NPs as well as subcutaneous administration of NPs into animals and subsequently analysis with flow cytometry after isolation of inguinal lymph nodes for determining the fluorescence intensity of antigen in CD19^+^B220^+^ B cells and F4/80^-^CD11b^+^CD11c^+^ DCs. They found that CD40-targeted PLGA nanovaccine (HPV E7/TLR2L/TLR3L), with 246 nm particle size and -28.5 mV zeta potential, were efficiently taken up by DCs both *in vitro* and *in vivo* and improved the maturation of DCs. The PLGA-CD40-based nanovaccine enhanced the proliferation of CD4^+^ T-cells and increased IFN-γ levels. Compared with non-targeted nanovaccines, CD40-targeted ones notably enhanced E7-specific CD8^+^ T-cells, leading to tumor growth suppression.^58^ Incorporation of antibodies and ligands on nano-sized vaccines can target APCs, such as DCs, for enhancing their therapeutic efficacy. There is evidence that engineering nanovaccines for targeting c-type lectin receptors-expressing DCs, Clec9a^+^ DCs, mannose receptor (CD206)-expressing DCs, DEC-205-expressing DCs, SR-B1^+^ DCs, TLR4-expressing DCs, and CD141^+^ DCs, stimulate potent CTLs to kill tumor cells.^14^

###  Liposomes

 Liposomes are safe, highly effective, and adaptable that can be engineered with preferred characteristics by controlling lipid composition, surface properties, size, morphology, charge, etc., making them flexible delivery systems. They are likely delivery systems stated to improve the immunogenicity of antigens in vaccines against cancer and have been used as delivery vehicles for antigens, adjuvants, DNA, and siRNA. It has been shown that both lipophilic and hydrophilic antigens can be loaded into liposomes in which the lipophilic cargos are incorporated into the lipid bilayer by chemical bonding or adsorption, whereas the hydrophilic ones are entrapped within the aqueous interior.^59,60^ There is evidence that the surface charge of liposomes is a critical parameter in the stimulation of immune responses. Positively charged liposomes are more efficiently taken up by DCs and APC-like macrophages, thus eliciting stronger CD8^+^ T-cells responses compared with negatively charged ones. Indeed, cationic liposomes are powerful carriers for subunit vaccines, stimulating potent immune responses at low doses.^61,62^ Mechanistically, the electrostatic interaction between cationic liposomes and negatively-charged cell membrane of APCs leads to the absorption of liposomes on APCs and subsequently the release of nanovaccine components into the cytosol of APCs.^62^ Some liposome-based vaccines are now commercially available, including Inflexal^®^ (for influenza), Epaxal^®^ (for hepatitis A), Mosquirix^®^ (for malaria), and Shingrix® (for Varicella zoster virus).^63^ Despite their advantages and great efforts to design liposomal-based nanovaccines, their application is faced with some challenges, including high cost, entrapment efficiency, and long-term stability.^64^ In addition, liposomes could activate complement system and induce complement activation–related pseudoallergy (CARPA), an acute hypersensitivity syndrome, resulting in cardiopulmonary distress, chills, headache, facial swelling, facial flushing, and anaphylaxis.^65^ Table 2 summarizes the application of liposomes and nanocomplexes as nanovaccines in the treatment of cervical cancer.

**Table 2:** Liposomes and nanocomplexes as nanovaccines in the treatment of cervical cancer

**Strategy**	**Nanostructure**	**Size/Charge**	**Antigen/Adjuvant**	**Therapeutic effects**	**Ref**
Liposome	DOTAP liposome and E7-lipopeptide	103 nm +44.5 mV	HPV16 E7 NA	↑CTLs, IFN-γ ↓Tumor growth	^66^
	DOTAP liposome and E7 peptide	NA NA	HPV16 E7 CpG, Poly I:C, and Cramp	↑Memory T-cells, CD8^+^ T-cells, IFN-γ ↓Tumor growth	^67^
	VacciMax® liposome and FP	NA NA	HPV16 E7 CpG	↑CD8^+^ T-cells, IFN-γ, CD84^+^ T-cells ↓Tumor growth	^68^
	Liposome and HA/E7 peptide	76 to 89 nm -51 to -63 mV	HPV16 E7 MPLA	↑Th1 cells, IFN-γ, IL-13 ↓Tumor growth	^69^
Nanocomplex	Chitosan NPs and E7 DNA	70 nm +20 mV	HPV16 E7 IL-12	↑CTLs, IFN-γ, IL-4 ↓IL-10, Tumor growth	^70^
	PPS NPs and E7 SLP	30 nm NA	HPV16 E7 CpG	↑ CD8^+^ T-cell/Treg ratio ↓Tumor growth	^71^
	Ad or Alb NPs and E7 peptide	Ad (123 nm, +8 mV) Alb (128 nm, +12 mV)	HPV16 E7 Ad or Alb NP	↑CTLs, IFN-γ, IL-10 ↓Tumor growth	^72^

 Regarding cervical cancer, various studies investigated the therapeutic activities of antigen and/or adjuvant-formulated liposomes.^73-75^ Zhao et al designed a liposome-based nanovaccine in which HPV16 E7 peptide and CpG ODN adjuvant were encapsulated into mannose-modified liposomes to elicit immune responses in TC-1 grafted tumor model. The Lip E7/CpG nanovaccine exhibited 122 nm particle size and +15 mV zeta potential, as well as 55% and 42% entrapment efficiency for E7 and CpG, respectively. This formulation not only decreased numbers of immune inhibitory cells such as macrophages and MDSCs, but also increased the populations of CD4^+^ and CD8^+^ T-cells, and IFN-γ-producing cells in tumors and spleens, and promoted CTL responses. In addition to modulatory effects on immune cells and responses, the Lip E7/CpG nanovaccine also inhibited tumor angiogenesis and tumor growth without any remarkable damage to the major organs of the vaccinated mice.^76^ Surface modification using mannose targets liposomes toward APCs because of the high expression of mannoses receptor (MR) on DCs and macrophages.^77^ Due to self-adjuvant activity of lipid moieties in lipoproteins and their ability to promote DCs maturation, Shen et al prepared a lipidated HPV E7 inactive mutant (rlipoE7m) lipoimmunogen plus 1,2-dioleoyl-3-trimethylammonium-propane (DOTAP) liposome-encapsulated native phosphodiester CpG (POCpG/DOTAP) formulation to target DCs and improve immune responses against tumors. Following characterization of nanovaccine preparation and loading efficiency, subcutaneously injection of TC-1 cells into the left flanks of naïve C57BL/6 mice two weeks before immunization was used to assess therapeutic antitumor effects. To analyze tumor-infiltrating cells, such as T-cells, myeloid cells, and DCs, TC-1 tumors were dissected, cutted into small pieces, and filterized to obtain single cells. The gathered single cells were stained anti-CD11c, anti-F4/80, anti-Gr-1, anti-CD11b, anti-CD45, Foxp3, CD25, anti-CD4, and anti-CD8 antibodies. Intravenous administration of rlipoE7m plus POCpG/DOTAP promoted IL-12 production by activating both conventional and plasmacytoid DCs, resulting in potent CTL and antitumor immune responses. Additionally, the production of IL-10 by DCs following immunization with rlipoE7m plus POCpG/DOTAP regimen dramatically reduced the number of tumor-infiltrating Tregs.^78^ To induce potent immune responses against HPV16 E7 SLPs, He et al constructed liposomal formulation incorporating cobalt–porphyrin–phospholipid (CoPoP) owing to its adjuvanticity activities. Besides the prophylactic efficiency, the formulated nanovaccine exhibited higher stability in serum and remarkably enhanced antigen delivery through internalization into immune cells, leading to strong infiltration of CD8^+^ T-cells within the TME and suppression of tumor growth.^79^ Another liposome-based, Poly(I:C) containing, and CD8^+^ T-cell-inducing adjuvant is CAF09 which Korsholm et al used the adjuvanticity of liposomes to design a nanovaccine. In the structure of CAF09, the main component of the liposome was dimethyldioctadecylammonium (DDA) and monomycoloyl glycerol (MMG)-1 was used as a stabilizer. Methodologically, the nanovaccine firstly characterized by particle size and zeta potential and then was used to subcutaneously immunize mice (2-3 times with two-week intervals). To assess T-cell responses, single-cell suspensions of splenocytes were restimulated with antigen and stained with antibodies to detect specific T-cell markers. They concluded that CAF09 stimulates cross-priming and CD8^+^ T-cells against peptide antigens and induces CTL responses. Interestingly, the optimal CD8^+^ T-cell responses using the CAF09-adjuvanted nanovaccine were observed in the intraperitoneal administration route versus the subcutaneous route. They also indicated that the HPV16 E7 peptide formulated in CAF09 protected 100% of mice against tumor challenge in the prophylactic model and remarkably reduced tumor growth in 38% of E7-expressing TC-1 tumors in mice.^80^ It is worth noting that the route of administration and system of administration also affects the response to nanovaccines. For instance, van der Maaden et al showed that although intradermal vaccination is able to induce effective immune responses in cancer immunotherapy, using a digitally controlled hollow microneedle injection system (DC-hMN-iSystem) compared with classical intradermal immunization with a syringe and hypodermic needle for delivering SLP-formulated liposomes could be more efficient in inducing T-helper and CTL responses.^81^ Efforts to formulate liposome-based nanovaccines against HPV-induced malignancies led to the translation of the PDS0101 vaccine to clinical trials (NCT05232851).

###  Nanocomplexes

 Antigen and adjuvant mixing is used to construct antigen-adjuvant structures on a nanoscale, called nanocomplex platform. Compared with NPs and encapsulation strategies, nanocomplex platforms have the advantage of antigen availability and faster release, leading to the induction of strong immune responses. Moreover, these platforms provide a cost-effective and facile strategy for developing nanovaccines. Despite the advantages, the nanocomplex formation strategy faces some challenges, including disassociation of antigen and adjuvant before receiving to the target site and alteration in the nature of antigen and adjuvant during modifications.^82^

 Saleh et al constructed an NP using MPG peptide to deliver HPV16 E7 DNA and assessed its therapeutic efficacy. MPG is an amphipathic and cell-penetrating peptide with 27 residues and three domains that constructs stable non-covalent NPs with nucleic acids. The domains of MPG peptide include an N-terminal hydrophobic domain derived from the fusion sequence of HIV gp41(GALFLGFLGAAGSTMGA) which is essential for cellular uptake, a hydrophilic and lysine-rich domain derived from the nuclear localization sequence of the simian virus 40 (SV40) large T-antigen (KKKRKV) which simplifies localization of negatively-charged cargo into the target cells, and an internal linker sequence (WSQP) which provides integrity and flexibility to flanking domains. They used ratios of basic amino acid residues in the MPG peptide to DNA phosphates (N/P ratio) for the construction of peptide/DNA complexes and assessed the stability of the complex using treatment with DNase I. At an N/P ratio of 10:1, the nanocomplex showed a spherical and regular shape in nano-sized range and protection against DNase I degradation. The MPG/E7DNA NPs derived from peptide/DNA complex induced strong IFN-γ response without substantial difference in IL-4 level and effectively inhibited tumor growth in TC-1 bearing mice. This study also emphasized that the size of NPs affects the immune responses, in which NPs induced cellular immune responses while microparticles stimulate humoral immune responses.^83^ Regarding NPs size, small-sized NPs can greatly pass through biological barriers and reach and accumulate in the lymph nodes, leading to uptake by lymph node-resident APCs.^84^ There is evidence that NPs with >100 nm in size are captured by APCs in the injection site and transported to lymph nodes, NPs with 20-100 nm in size drain into the lymph nodes and are taken up by lymph node-resident APCs, and NPs with <20 nm in size are eliminated after drain to blood capillaries.^85,86^ Moreover, NPs with >500 nm in size are internalized into the cells through phagocytosis, whereas endocytosis is the main mechanism for cellular uptake of NPs with 20-200 nm in size.^87^ Studies revealed that small particles could induce Th1 and CD8^+^ cells compared with particles with larger sizes which promote Th2 responses.^41^ Wang et al took advantage of SpyTag/SpyCatcher for delivering HPV16 E7 epitope and MC38 neoantigen to DCs. They showed that the conjugation of antigen and/or neoantigen to the SpyTag/SpyCatcher system on ferritin NPs efficiently enhanced the capture of NPs by draining lymph nodes, leading to stronger CTL responses and suppression of tumor growth. Additionally, they immunized TC-1 tumor-bearing mice with the combination of ferritin-E7_(43-62)_ NP vaccine (1 nmol, three times at 5 days interval) and anti-PD-1 (50 μg, on days 12, 15, and 18). The combination of nanovaccine with PD-1 checkpoint blockade exhibited superior anti-tumor activities.^88^ In another study, Tang et al used the cell-penetrating peptide HIV-1 Tat_49-57 _in fusion with HPV16 E7_49-57_ epitope and the granulocyte-macrophage colony-stimulating factor (GM-CSF) DNA to construct a self-assembled NP for evaluating its therapeutic efficacy. In this system, peptides protect DNA from hydrolysis by DNase I. The nanovaccine showed efficacy in both prophylactic and therapeutic strategies through the induction of long-term survival and reduction in tumor growth. Mechanistically, the Tat-E7/pGM-CSF nanovaccine promoted E7-specific and CD8^+^ T-cells as well as CTL immune responses.^89^ It is worth noting that the positively charged peptides, such as Tat, interact with negatively charged surface proteoglycan, leading to the induction of higher endocytosis. To take advantage of this electrostatic interaction on the cellular surface, Hashemi Goradel et al designed an experiment to change the negative charge of three nanoadjuvants to a positive one and enhance their therapeutic activities. To this end, they incubated positively charged HPV16 E7_49-57 _with three negatively charged adjuvants, including adenovirus (Ad), CpG-ODN, and aluminum phosphate (AlPO_4_). In this strategy, the charge of nanoadjuvants was changed from negative to positive due to the formation of a complex with positively-charged E7 epitope, without remarkable change in their size. The intratumoral administration of E7/nanoadjuvants regiments revealed that heterologous administration of nanovaccines elicited stronger immune responses compared with homologous regimens and naked nanoadjuvants, characterized by higher levels of IFN-γ, IL-10, and CD107, as a marker of CTL responses, as well as tumor growth inhibition. Interestingly, they reported that the sequence of nanovaccine administration also affects anti-tumor activities. For instance, priming with Ad/E7 nanocomplex and boosting with AlPO_4_/E7 nanocomplex exhibited more efficiency than the vice versa regimen.^90^ Therefore, it could be considered that in addition to constructing strategies and platforms, the sequence and planning of administration also are pivotal for the therapeutic activities of nanovaccines. Regarding the effect of sequence and time of administration on anti-tumor activities in cancer immunotherapy combinations, it has been shown that vaccination prior to immune checkpoint inhibitors (ICIs) promote baseline immunity and help to convert the “cold” TME to the “hot” one, leading to superior response to ICIs.^91,92^ Kim et al revealed that sequential treatment with small lipid nanoparticle (SLNP)-based nanovaccine and anti-PD-1 was more effective in inducing a potent immune response and inhibiting tumor re-growth compared with simultaneous treatment strategy.^93^ Unfortunately, there are limited studies to explore the role of administration sequence and this field requires more attention.

## Conclusion

 Among various approaches to cancer immunotherapy, vaccines have attracted attention and several platforms have been developed, including nucleic acid-based, protein/peptide-based, vector-based, and cell-based vaccines. Despite enormous efforts in designing and constructing cancer vaccines, they encounter several challenges, such as poor stability in blood, poor delivery *in vivo*, complications, high cost of treatment, tumor immune escape, and low immunogenicity. To overcome the limitations, nanovaccines with promising efficiency have emerged for the treatment of cancers, including cervical cancer.

 In nanovaccine platforms, antigens and/or adjuvants are encapsulated into the NPs or conjugated on the surface of nanostructures to deliver immune-promoting agents to APCs or tumor cells. Indeed, nanovaccine can bypass poor antigen presentation and immune evasion in the treatment of cancers by providing sophisticated delivery systems. It could be considered that the effectiveness of nanovaccines is frequently determined by several parameters, such as safety, stability, kinetics, geometry, biocompatibility, hydrophilic property, charge, and nanomaterial size. Moreover, precise targeting of tumor cells not only enhances the therapeutic efficacy of nanovaccines, but also minimizes systematic toxicity. Nano-based vaccines also provide therapeutic combination potential with other cancer immunotherapy agents, such as immune checkpoint inhibitors and chimeric antigen receptor (CAR) T-cells. For instance, tumor cells could induce T-cell anergy following vaccination with upregulating immune checkpoints and triggering the programmed death ligand 1 (PD-L1)/PD-1 pathway, in which a combination of nanovaccine with immune checkpoint inhibitors blocks the PD-L1/PD-1 pathway, leading to a decline in immune evasion and boost in immune responses. The combinational treatment strategy also engages different parts of the immune system to use the overall potential of the body to elicit strong responses against tumors. Thus, the combinational approach not only amplifies more robust, diverse, and sustained immune responses against tumor cells, but also overcomes the immunosuppressive TME.

 Bioinformatics analyses as well as next-generation sequencing could help generate and select tumor-specific and personalized potential antigenic epitopes which could be employed for designing nanovaccines to induce potent immune responses against cervical cancer. Furthermore, self-assembled structures by chemical conjugation of the epitope to a self-assembling peptide or the construction of amphiphilic peptide conjugates hold great promise as next-generation nanovaccines to induce more potent cellular responses, leading to a remarkable increase in the therapeutic efficiency of peptide-based vaccines.

 Despite undeniably promising properties and therapeutic potential in the treatment of cancer, there are some challenges and limitations in the development of nanovaccine and their translation into the clinic. A primary public concern surrounding the development of novel nano-based vaccines and adjuvants is safety. While limited research suggests that inorganic NPs may exhibit inherent toxicity following prolonged exposure, their cytotoxicity is often dose-dependent. Given the relatively recent integration of NPs into medicine and the absence of a comprehensive safety profile in human vaccinations, it is imperative to conduct extensive safety assessments in animal models prior to human trials, ensuring the safety of the materials and adjuvant properties before their clinical application. Designing reproducible and highly precise nano-sized vaccines requires complex manufacturing procedures as well as highly cost production and regulatory processes. For example, the development of TME-responsive nanovaccines to adapt to the specific conditions of the TME for releasing their cargo in the targeted site could be considered a promising strategy to enhance immune responses against cancer, however, tumor heterogeneity acts as an obstacle to producing an inclusive nanovaccine for all patients. Moreover, variations in NP physicochemical properties, including surface charge, size distribution, antigen/adjuvant release profiles, and conjugation/encapsulation efficiency, may trigger unintended non-specific immune responses upon biodegradation. Additionally, while small-scale laboratory research facilitates the scalability of nanovaccine production, large-scale sterile manufacturing poses significant challenges. Today, coaxial turbulent jet mixer and particle replication in non-wetting templates (PRINT) technologies are available for scaling up NPs and producing adjustable, reproducible, and uniform NPs, respectively. The absence of clear regulatory guidelines for nanovaccine production introduces uncertainty for vaccine developers. Regarding the excellent results of preclinical studies and the disappointing results in clinical trials, differences in immunology and cancer biology between human and animal models could be considered. Generating genetically engineered mouse models, using different technologies such as clustered regularly interspaced palindromic repeats (CRISPR)/Cas9, and humanized mouse models instead of conventional transplantable models to mimic the natural tumor development and the TME will help translate preclinical results to clinical trials.

 Integrating multi-omics approaches, including immunological profiles, proteomics, and genomics, will direct the design of nanovaccine formulation toward comprehensive, reliable, and precise strategies. For instance, using single-cell sequencing technology will provide in-depth knowledge of the interaction between immune cells, such as APCs, and nanostructures, suggesting the proper NPs, adjuvants, and antigens for the development of personalized nanovaccines. To reach personalized nanovaccines, computational methods, such as artificial intelligence and bioinformatics, will be helpful. Additionally, shifting from the polymeric NPs towards biomimetic nanovaccines, such as bacterial outer membrane vesicles, virus-like particles, and cell membrane decorated NPs, with multi-antigenic and inherently immuno-stimulatory properties will provide safe and more potent strategies in the field of cancer therapy. Furthermore, developing nanovaccines with needle-free delivery systems without needing ultra-low temperature cold chains will improve the accessibility and safety of vaccines. One of the main limitations of nanovaccine development is the lack of predictive biomarkers. Determining and identifying biomarkers as patient-specific signatures will help predict treatment outcomes with nanovaccines. Thus, translating a nanoformulated vaccine from preclinical into clinical practices demands addressing reproducibility, efficacy, safety, regulatory, and long-term toxicology concerns. Taken together, innovations in personalized medicine, bioinformatics, immune profiling, and engineering will shape the future of nanovaccines to develop reliable anti-cancer agents according to the needs of patients.

## Competing Interests

 The authors declare no conflict of interest.

## Data Availability Statement

 No data was used for the research described in the article.

## Ethical Approval

 Not applicable.
